# Valorization of Aluminum Dross with Copper via High Temperature Melting to Produce Al-Cu Alloys

**DOI:** 10.3390/ma14154117

**Published:** 2021-07-23

**Authors:** Artur Kudyba, Shahid Akhtar, Inge Johansen, Jafar Safarian

**Affiliations:** 1 Department of Materials Science and Engineering, Norwegian University of Science and Technology (NTNU), Alfred Getz Vei 2, 7034 Trondheim, Norway; jafar.safarian@ntnu.no; 2 Hydro Aluminum, Romsdalsvegen 1, 6600 Sunndalsøra, Norway; shahid.akhtar@hydro.com (S.A.); inge.johansen@hydro.com (I.J.)

**Keywords:** Al dross, wettability, white dross, contact angle, aluminum recovery, sessile drop

## Abstract

The valorization of aluminum dross for Al recovery was performed via its mixing with metallic copper to produce Al-Cu alloys. This approach was with the intention of establishing a new smelting process to treat the dross with Cu scrap use. To evaluate the high temperature interaction of the materials, the wettability of a Cu-containing aluminum alloy with the non-metallic components of the dross was studied by the sessile drop method. It was found that the wetting was weak via temperature changes at 973–1373 K, and consequently no proper metal separation occurred. To better separate the metallic and non-metallic phases with larger density differences, a higher Cu portion was considered to obtain a significantly denser metallic phase, and it was found that partial separation of the Al in an Al-Cu alloy is possible. The complete separation of the metallic components of the dross was, however, experienced by the dross and copper melting with the addition of pre-melted calcium aluminate slags at elevated temperatures. It was found that Al-Cu alloys were produced and separated from the adjacent slags, and the aluminum oxide of the dross ended up in the slag phase. Moreover, the characteristics of the produced slags depend on the process charge.

## 1. Introduction

Aluminum alloys are light, conductive, and corrosion resistant, properties that have made them a widely used material with applications in the aerospace, architectural construction, and marine industries, as well as in many domestic contexts [1,2]. Pure aluminum is the Earth’s third most abundant element, accounting for about 8% of the mass of the Earth’s crust, and it never occurs as a free element in nature [3,4]. At present, aluminum is mainly produced via two different methods: (I) a primary aluminum production from bauxite ore via the Bayer process for alumina extraction, followed by Hall–Heroult electrolysis for Al extraction from alumina, and (II) by recycling aluminum from process scrap and used aluminum products [3,4,5,6,7]. The world’s production of aluminum is increasing every year and was approx. 64 million metric tons in 2019 with a daily average of 174.5 thousand tons [3,7], while in 2020 it was over 65 million metric tons in 2020 [8]. As a result of the exposure of liquid aluminum to the oxidizing atmosphere that is present during the process of melting and alloying, a surface oxidation takes place, leading to the formation of a semisolid skin over the molten Al metal, which also hinders further oxidation [7]. This floating skin over liquid Al is called aluminum dross and consists mainly of aluminum oxide, metallic aluminum, magnesium spinel (MgAlO_4_), periclase (MgO), quartz (SiO_2_), and salts with small traces of aluminum carbides and nitrides [9,10]. The recycling of Al dross, which is a by-product, is important from both environmental protection and economic points of view [4,6]. Generally, there are two types of Al dross: (I) White Dross (the primary dross) and (II) Black Dross (the secondary dross) [3,7].

Aluminum White Dross (AWD) forms during the primary production of aluminum and contains a high percentage of Al and Al_2_O_3_ i.e., around 75% Al, and below 5% of salts [3,11]. Aluminum Black Dross (ABD) consists of a low amount of metal substance and is formed at secondary aluminum production routes. ABD involves a mix of aluminum oxides and slag, with recoverable aluminum content is in the range 12–18% and a greater amount of salt substance; for instance, greater than 40% stood out from white dross [11,12,13,14,15,16]. Each year, the world’s aluminum industry produces approximately four million tons (Mt) of AWD [3,14]. More than a million tons of ABD are reported throughout the world each year, and around 95% of this material, which is hazardous, is landfilled [3,4,17]. Aluminum dross is a potentially toxic industrial waste inevitably generated in aluminum smelter plants. The safe disposal of Al dross as a waste is a problem for the aluminum industry because of the effects of its improper disposal on the eco-system. Owing to the large annual production of Al dross and its environmental and economic impacts, aluminum dross undergoes industrial treatments to extract valuable products, including metallic aluminum [7]. Basically, two methods of Al dross treatment are used: (I) a pyrometallurgical, which is a conventional method of treating Al dross, liberating metallic aluminum in the liquid state; and (II) a hydrometallurgical, which involves an extraction of metallic aluminum from the Al dross by converting it into aluminum salts and compounds [3,7]. The metal extraction using pyrometallurgical process yields a good metal recovery rate. In the case of lower metallic content in dross, the hydrometallurgical process is preferred [18]. The recycling of aluminum dross is crucial for environmental protection, economic reasons, and sustainable development regarding circular economy [7]. 

**Figure 1:**
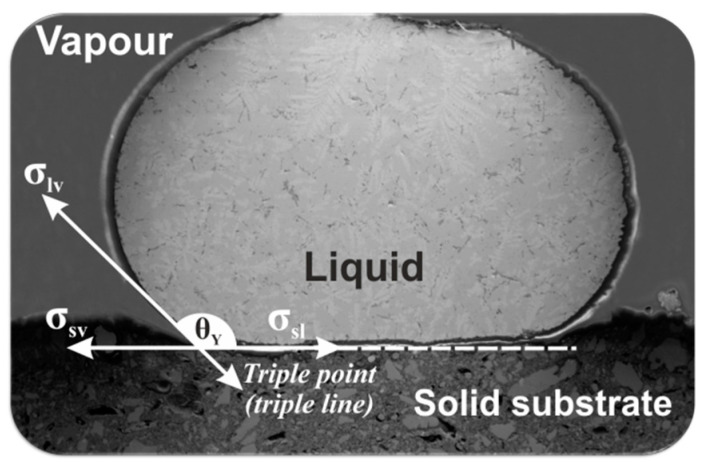
A scheme showing the concept of Young’s contact angle θ_Y_ for a sessile drop contacting a solid surface.

The main purpose of the present work was to experimentally examine the effect of copper addition into AWD on the Al recovery from the white dross via a high temperature melting process with and without flux addition. In this case, the melting of AWD was with the goal of recovering the metallic Al in AWD in the form of Al-Cu alloys. Moreover, the wetting behavior of a liquid Al-Si-Cu commercial alloy in contact with the non-metallic components of AWD (solid Al dross substrate) was studied to obtain a proper understanding about the mechanisms of high-temperature interaction between liquid aluminum alloys with the non-metallic components of the AWD. A full understanding of this mechanism will allow for the effective separation of the metallic phase from the Al dross material (mainly oxide phases), which may contribute to more effective recycling of aluminum dross. The wetting and spreading behavior of liquid aluminum alloy on a surface of solid Al dross substrate is an important phenomenon that should be considered for improving the recycling of aluminum dross. Generally, the wetting of a solid by a liquid depends on the involving interfacial tensions between the three involved phases: solid/vapor (SV), solid/liquid (SL), and liquid/vapor (LV) [19]. A value of Young’s contact angle *θ_Y_* (Figure 1) is an ideal quantity, theoretically defined [20] from the mechanical equilibrium calculated for a liquid droplet contacting a substrate under the action of horizontal forces acting at the three-phase contact line (a so called triple line (TL)). These forces arise from the interfacial energies of the liquid/vapor σ_lv_, solid/vapor σ_sv_, and solid/liquid σ_sl_ interfaces [21]. Consequently, these considerations are summarized by the Young equation [20]:

σ_sv_ − σ_sl_ = σ_lv_ cosθ, (1)

A detailed description of Young’s equation, the sessile drop method, and corresponding testing procedures are reviewed and available in Refs. [22,23].

## 2. Experimental Procedure

The materials preparation and applied methodology are described as follows.

### 2.1. Materials and Sampling

#### 2.1.1. Wettability Test

The substrate for the wettability tests was performed from a commercial AWD (Hydro Aluminum, Sunndalsøra, Norway) with a particle size below 1 mm. AWD was first collected from the skimmed dross over the surface of molten primary Al and an Al-Si-Mn alloy (grade series 4000, respectively). To directly extract a representative Al dross sample, a special sampling tool was introduced into a dross tub, and when the Al dross was skimmed from the reverberatory furnace into the tub in which the tool was positioned in, a portion of the hot dross was collected. The aluminum dross was subsequently subjected to a ball milling, and a detailed description of this procedure is reviewed in Refs. [3,7]. After the milling process, the milled product was fractioned by sieving, and the material from the smallest fraction with a particle size below 1 mm was used to create the substrates as it has the majority of the non-metallic components of the AWD [3,7]. The metal sample used in the wettability tests was an alloy of Al-5.6%Si-7.6%Cu that was created by mixing a commercial Al-Si-Cu alloy (92.6 wt.% Al, 6.7 wt.% Si, 0.47 wt.% Cu, 0.35 wt.% Mg, 0.15 wt.% Fe, 0.09 wt.% Mn) with pure copper (99.99%).

#### 2.1.2. Melting Trials 

The above AWD was used for the melting tests; however, a particle size of 2–10 mm was used. In experiment 1, only the dross particles and Cu particles were mixed, with no flux addition. In experiments 2 and 3, a CaO-Al_2_O_3_ synthetic-slag flux was added. Two different slag compositions were initially prepared: CaO/Al_2_O_3_ = 2 (experiment 2) and CaO/Al_2_O_3_ = 4 (experiment 3) via mixing pure CaO and Al_2_O_3_ powders (above 99% purity level), and then melting them in a top-open induction furnace at 1873 K in a graphite crucible (Svenska Tanso AB, Jönköping, Sweden) for 1 h. For the AWD melting trials, metallic Cu (99.99%) particles were added with sizes of 1–5 mm, and the charge mixture details are given in Table 1.

### 2.2. Melting Procedure

#### 2.2.1. Wettability Test

**Figure 2:**
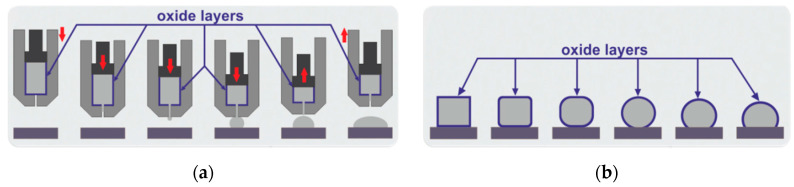
A scheme of the high-temperature wettability test by classical Sessile Drop method (SD) combined with procedure: (**a**) Capillary Purification (CP); (**b**) Contact Heating (CH) [21,22,23,24,25].

The wettability experiment of the Al-Si-Cu/Al dross couple was carried out by the classical Sessile Drop (SD) method combined with a newly introduced Capillary Purification (CP) procedure (Łukasiewicz Research Network—Krakow Institute of Technology, Kraków, Poland, Figure 2a) that allows for the purification of the reactive molten metal drop from its surface oxide layer. The native oxide film was mechanically removed during the squeezing of the liquid Al alloys-drop from the capillary (Figure 2a), which allowed for the measurement of true contact angle values. The Al-Si-Cu/Al dross couple was subjected to an SD experiment that was performed using the experimental device (for investigation of high-temperature capillary phenomena of liquid metals and alloys) described elsewhere [24,25].

**Figure 3:**
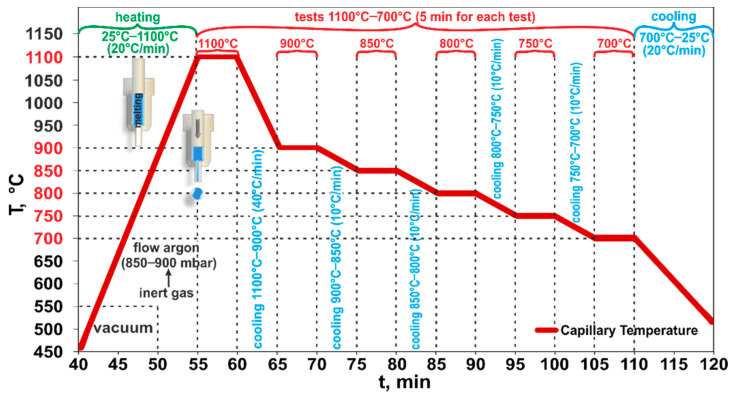
Temperature profile of the high-temperature wettability test of the Al-Si-Cu/Al dross couple carried out by a step-cooling with six intervals.

The wettability experiment included 6 intervals at 1373 K/5 min, 1173 K/5 min, 1123 K/5 min, 1073 K/5 min, 1023 K/5 min, and 973 K/5 min. The main purpose of this test was to examine the effect of testing temperature on the wetting characteristics and to establish applicability limits of selected materials. The exact diagram of the temperature profile used in the wettability experiment is shown in Figure 3.

After placing the alloy/substrate couple inside the chamber, gases were evacuated using Scroll (Duo 10M, Pfeiffer Vacuum Gmbh, Asslar, Germany) and turbo-molecular pumps (TW 701, Leybold Vakuum Gmbh, Köln, Germany). When pressure inside the chamber reached the value of about 1 × 10^−6^ mbar, heating was started at a rate of 293 K/min to test temperature 1373 K (Figure 3). At a temperature of 823 K, the inert gas (flowing argon, *p* = 850–900 mbar) was introduced to the chamber to suppress the evaporation from the Al-Si-Cu alloy. During the experiments, the Al-Si-Cu/Al dross couple images were recorded by a high-speed digital CCD camera (Microtron MC 1310, Microtron GmbH, Unterschleißheim, Germany) at 50 frames per second, and then the images were processed to measure the change of contact angle values over time and to analyze the related wetting kinetics by using ASTRA 2 software. After the high temperature tests, the solidified couple was removed from the chamber and subjected to a structural characterization of the prepared cross sections by using the Zeiss Ultra 55 Scanning Electron Microscope (SEM, Carl Zeiss Microscopy GmbH, Jena, Germany), coupled with energy-dispersive X-ray spectroscopy (EDS, Carl Zeiss Microscopy GmbH, Jena, Germany).

#### 2.2.2. Melting of Al Dross with Cu and Synthetic Slag

**Figure 4:**
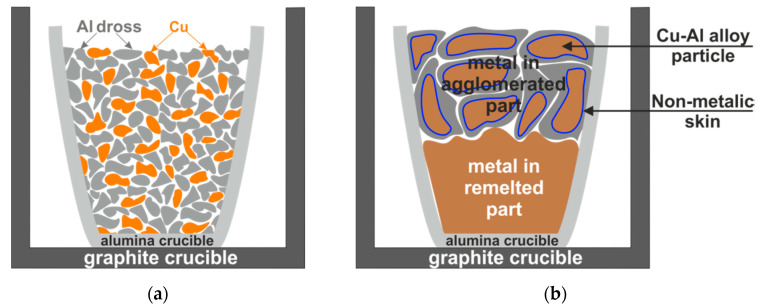
A scheme of (**a**) charged materials for experiment 1 in-crucible before the reaction test, and (**b**) the produced metal in agglomerated and remelted parts after the reactions.

In experiment 1, Al dross was mixed with pure Cu in the mass proportions of 40% and 60%, respectively. The mixture was charged into an alumina crucible and then in a graphite crucible, as schematically shown in Figure 4a.

The mixture in the crucible was subjected to annealing at a temperature of 1373 K for 30 min in a top-open induction furnace (Cold Crucible, NTNU, Trondheim, Norway) described elsewhere [3,7] in a protective atmosphere (flow gas Ar 5.0 at a constant pressure of 1030 mbar), resulting in melting of the metallic components. After melting, the furnace power was turned off and, consequently, the molten components solidified. The remelted and separated metal moved to the bottom of the crucible, while the agglomerated part remained above it, as schematically illustrated in Figure 4b. The produced remelted metal and the agglomerated part were separated after breaking the crucibles. Metallography samples were then created from them by mounting in epoxy resin followed by grinding and polishing. The structure and chemical composition of different phases were determined by using the same SEM/EDX mentioned above.

**Figure 5:**
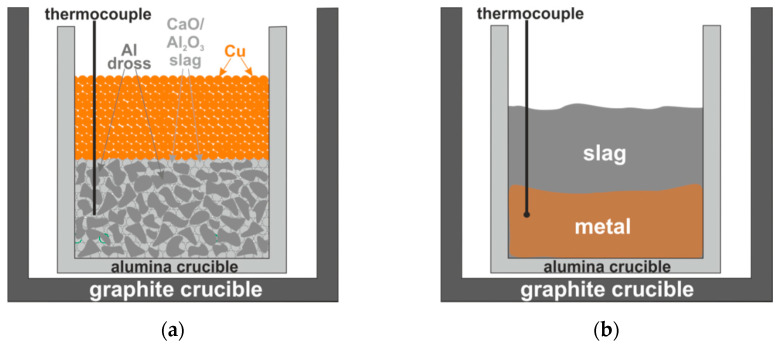
A scheme of (**a**) charged materials for experiments 2 and 3 in-crucible before the reaction test, and (**b**) the produced slag and metal after the reactions.

For experiments 2 and 3, Al dross was mixed with CaO-Al_2_O_3_ synthetic slags (Table 1). The mixtures were first charged into alumina crucibles and pure Cu was deposited on the top of these mixtures, and then they were put in graphite crucibles as schematically shown in Figure 5a.

The crucibles with the charge mixtures were put in an induction furnace and a thermocouple was put into the charge mixture to measure the temperature inside the crucible. Each crucible was heated at about 293 K/min up to 1773 K and held for 30 min in a protective atmosphere (flow gas Ar 5.0 at a constant pressure of 1030 mbar). After melting, the furnace power was turned off and, consequently, the molten components solidified. The metal was much heavier than the slag and it sunk down to the crucible’s bottom, as schematically illustrated in Figure 5b. The produced metal and slag were separated after breaking the crucibles and subjected to a structural characterization by SEM/EDX. Moreover, the produced final slags from experiments 2 and 3 were studied by X-Ray Diffraction (XRD) analysis using a Bruker D8 A25 DaVinci X-ray Diffractometer with CuKα radiation with LynxEye™ SuperSpeed Detector (Bruker Corporation, Billerica, MA, USA) [26,27].

## 3. Results and Discussion

The obtained results are presented and discussed as follows.

### 3.1. Wetting Behaviour of Al Dross Substrate

**Figure 6:**
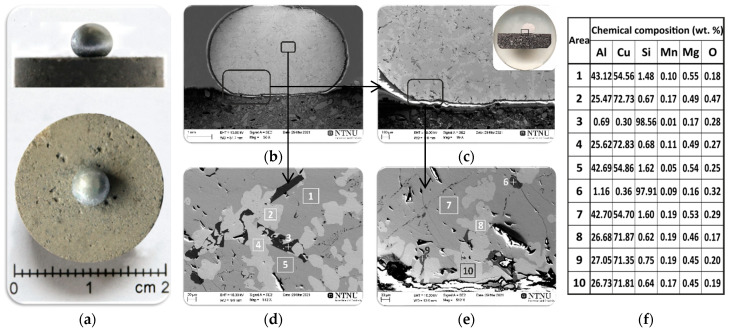
The macro views of Al-Si-Cu/Al dross couples after the wettability test (**a**) and the results of the SEM/EDS microstructural analyses of the cross sections of sample at magnification 30× (**b**); 89× (**c**); and 512× (**d**,**e**); EDS (**f**).

**Figure 7:**
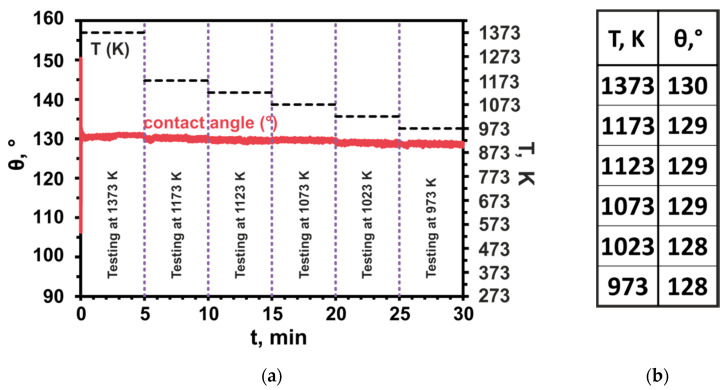
The results of high-temperature wetting behavior of Al-Si-Cu alloy/Al dross couple: (**a**) wetting kinetics curve (contact angle vs. time); (**b**) values of the contact angle after 5 min for six temperature intervals.

**Figure 8:**
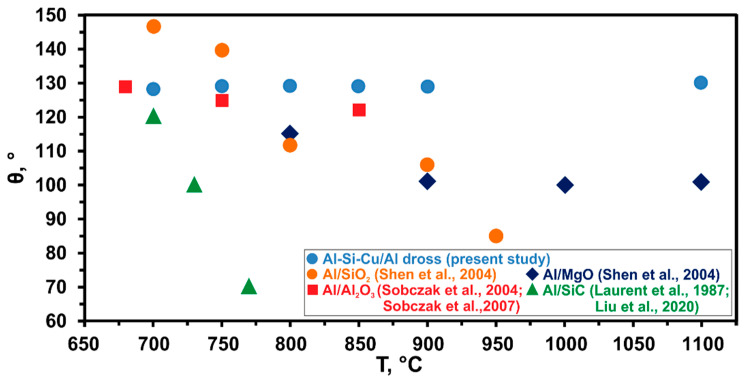
Comparison of the obtained values of the contact angle of the tested Al-Si-Cu/Al dross system with the reported in literature values of the Al/substrates systems, with a contact time of 5 min and at various temperatures [29,33,34,41,46,47].

Figure 6a shows macro views of the Al-Si-Cu/Al dross couple after the wettability test and the results of the SEM and EDS microstructural analyses of the cross sectioned sample (Figure 6b–e). A regular round shape of the Al-Si-Cu drop solidified on the Al dross substrate was observed. The solidified metal drop was located in the central part of the substrate and was characterized by a smooth and shiny surface devoid of the oxide layer (Figure 6a), which was the result of the CP procedure used in the wettability test. Typical SEM images from the metal/dross interfacial area are given in Figure 6b–e, and selected areas were analyzed by the EDS. The dark grey phase in the SEM images of Figure 6b–e shows the presence of a dominant phase of metal matrix in the drop that consists mainly of Cu and Al elements. The results of local chemical composition analyses by EDS from three areas 1, 5 (Figure 6d) and 7 (Figure 6e) revealed that it was composed of an average of 54.7 wt.% Cu and 42.8 wt.% Al. According to the Al-Cu phase diagram [28], the metal contained a matrix structure of θ-CuAl_2_ phase in these areas. The presence of a second dominant phase consisting of the Cu and Al elements was also found for the light grey phase that was analyzed by EDS analysis at four areas 2, 4 (Figure 6d), and 8, 10 (Figure 6e). The analysis revealed that it was mainly composed of an average of 72.3 wt.% Cu and 26.1 wt.% Al. Based on the results of the EDS evaluations and considering the Al-Cu phase diagram [28], it is concluded that the chemical composition of light grey phase corresponds to the ῃ′-CuAl phase. Moreover, a needle-like Si phase (point 3 in Figure 6d and point 6 in Figure 6e) was also identified, containing an average of 98.2 wt.% of Si; these could be primary Si particles that formed during the solidification of the Al-Si-Cu alloy. The identified small amount of oxygen could be due to the surface oxidation of the metal phase during cutting and sample preparation. An important result was that the commercial Al dross substrate did not give significant impurities to the metal sample.

The side view of the sample in Figure 6a shows that the dross had not been wetted by the Al alloy. Figure 7 shows the results of high-temperature wetting behavior of liquid Al-Si-Cu alloy in contact with Al dross substrate by applying the CP procedure at temperatures 1373, 1173, 1123, 1073, 1023, and 973 K, for 5 min at each of the temperatures. It was found that the contact angle values were very high in the whole examined temperature range and they slightly decreased from θ_1373_ = 130° to θ_973_ = 128° (Figure 7b), with decreasing testing temperature from 1373 to 973 K, respectively. The wetting kinetics curve of the studied Al-Si-Cu alloy/Al dross system was constant and showed a plateau at each of the tested temperatures (Figure 7a). Based on the analysis of the wetting kinetic curve (Figure 7a) and values of the contact angle after 5 min for six temperature intervals (Figure 7b), it can be concluded that the temperature change in the range of 1373 to 973 K does not affect the wettability of the Al-Si-Cu alloy/Al dross system, i.e., the value of the contact angle does not change. It should be noted that the contact angles measured after 5 min holding at each consecutive temperature cycle presented very high values and they were significantly higher than 90°, i.e., the wettability criteria, which proves that the Al-Si-Cu alloy/Al dross couple is a non-wetting system.

In all the former studies [29,30,31,32,33,34,35,36,37,38,39,40,41,42,43,44,45,46,47], no significant work was performed on studying the wetting behavior of liquid Al-Si-Cu alloy in contact with Al dross substrate with particular emphasis on the CP procedure. Hence, the novelty of the present study is first providing wettability information of Al-Si-Cu/Al dross systems, secondly the application of the CP procedure in the Al dross wettability test, and third providing microstructural information of Al-Si-Cu/Al dross systems. Literature survey [29,30,31,32,33,34,35,36,37,38,39,40,41,42,43,44,45,46,47] revealed that available papers are mostly concerned with the study of the wettability of pure Al [29,30,31,32,33,34,35,36,37] and its alloys [38,39,40,41,42,43,44,45] in contact with various types of substrates, such as Al_2_O_3_ [29,34], TiO_2_ [30,34], C [31,38,40,44], NiO [32], SiC [33,39], ZrO_2_ [34], CoO [35], ZnO [36], Y_2_O_3_ [37], SiO_2_ [46], and MgO [47]. In general, the wettability of Al and its alloys is better on carbon and carbides than on the oxides. Klinter et al. [42] analyzed the wetting behavior of Al-Cu alloys (Al-4Cu, Al-7Cu, Al-11Cu, Al-33Cu) on sapphire substrates by the SD method combined with a Contact Heating (CH) procedure (Figure 2b) at 943, 973, 1003, 1023, and 1073 K, in high vacuum. Since the native oxide film cannot be removed from Al-Cu drop using of the CH procedure, they had to increase the testing temperature up to 1073 K. By taking into account thermodynamic considerations, at such temperatures the oxide layer at the surface of the liquid Al-Cu drop was thermally removed. The authors reported that sapphire was not wettable by liquid Al-4Cu alloy in the temperature range 943–973 K and the decrease of the contact angle θ was observed from 122° to 92°, respectively. Further decreasing of the contact angle θ to the final value of 85° was observed by increasing the temperature up to T = 1073°C and after a holding time of 15 min. The same problem of the oxide layer on the surface of liquid Al-Si drop was described by Mao et al. [44], who analyzed the wetting behavior of Al-Si alloys (Al-6Si, Al-10Si, Al-20Si) on graphite substrates by the SD method combined with the CH procedure (Figure 2b) in the temperature range of 1023–1273 K, under a high vacuum atmosphere. They reported that at 1023 K the contact angle θ was very large, close to 160°, which corresponded to an oxidized surface surrounding the drop. Increasing the temperature to 1273 K caused the contact angle θ to decrease rapidly to about 124°, and then after 45 min at 1273 K the contact angle θ decreased to 86, 83, and 61° for the Al-6Si/C, Al-10Si/C, and Al-20Si/C systems, respectively. However, upon testing at temperatures much higher than the melting point of the tested materials, the substantial evaporation of the material should be carefully considered in order to limit the contamination of the experimental environment and to protect the apparatus against severe damage. In addition, a strong material evaporation increases the ratio of the solid base diameter to solid height which, in case of reactive phenomena at the interface, makes the chemical-physical meaning of the measured contact angle values “apparent” [25]. Figure 8 shows the comparison of the present work’s results with selected literature, and, as seen, the contact angle measurements in the present work were close to the Al/Al_2_O_3_ results [29,34]; this may be due to the existence of Al_2_O_3_ as the main non-metallic component in the utilized dross.

The above findings are very important to understand the mechanisms of high-temperature interaction between liquid Al-Si-Cu alloy/Al dross systems when the remelting of the dross is performed. Non-wetting of the liquid Al-Si-Cu alloy in contact with Al dross substrate will not allow the effective separation of the metallic phase (through accretion) from the remaining non-metallic part. It was previously observed that melting of separated Al-rich particles of the dross does not yield the complete separation of the Al [3]. Obviously, the low Al recovery may be due to the low wetting of Al by the non-metallic components of the dross as observed above. This low wetting causes the melted Al portion of the dross to be distributed in a solid or semi-solid nonmetallic matrix and with unsuccessful accretion to acquire a metal separated from the nonmetallic phase. In the slat-based remelting process, however, the use of salt improves the wetting properties as the non-metallic components of the dross. The molten salt interaction with the non-metallic components of the dross yields a low viscous molten/semi-molten phase. This causes low interfacial tensions between the molten salt and the molten Al, and hence the Al recovery is significantly enhanced. In the present work, however, a salt-free process for Al recovery from the dross was studied and, therefore, the melting trials were performed without any salt use.

### 3.2. Melting Behavior of Al Dross and Cu Mixtures

The melting of separated Al-rich particles of the dross was performed in two approaches via changing the physical properties in the system for both the metallic portion and the non-metallic portion. Therefore, melting was performed by Cu metal addition to change the overall metal composition significantly and to a high Cu-containing alloy. Moreover, additional CaO-Al_2_O_3_ slag flux was used to dissolve the non-metallic portion of the dross into a co-existing slag. These approaches were based on the results of wetting trials in Section 3.1, and further results from melting trials are presented and discussed as follows.

#### 3.2.1. Flux-Free Melting of Al-Rich Dross with Cu

**Figure 9:**
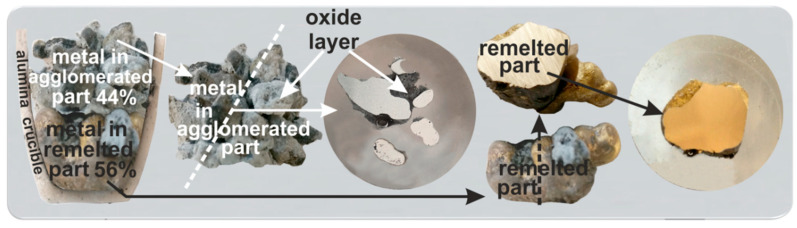
The broken crucibles after experiment 1 and the prepared sample for the SEM/EDX study.

Figure 9 shows macro views of the broken crucibles after the melting of the mixture of Al dross particles with pure Cu chips shown in Figure 4.

**Figure 10:**
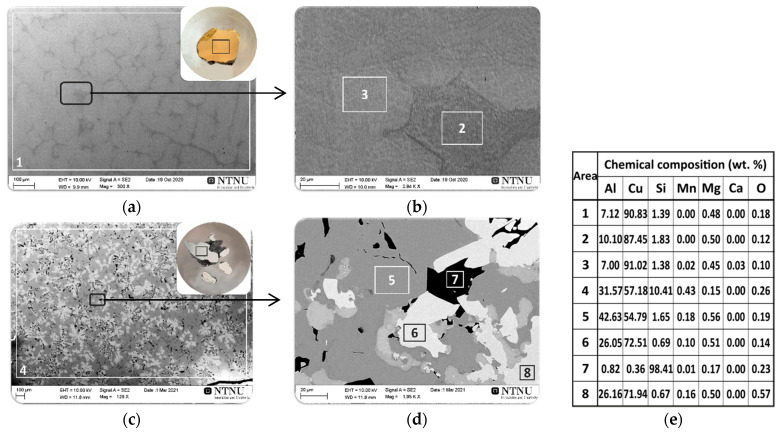
The SEM/EDS microstructural analysis of the cross-section of exp. 1 sample from remelted part (**a**,**b**) and agglomerated part (**c**,**d**); EDS (**e**).

Considering the macro views of the broken crucible after remelting, the division of the sample into two areas is possible, namely the area of the agglomerated part on top and the complete remelted part on the bottom. The percentage masses of the agglomerated and melted parts were determined to be about 44% to 56%, respectively. The induction melting of the mixture of Al dross with pure Cu contributed to homogenizing the structure of the completely remelted part, which consisted mainly of a metallic matrix of the copper-aluminum phase containing an average of 91 wt.% Cu and 7wt.% Al (areas: 1, 3 in Figure 10a,b respectively). Obviously, most of the melted Al droplets (surrounded by an oxide layer) in the dross remained in the top agglomerated part and could join the copper to form a single melt in the crucible. This might be attributed to the role of surface aluminum oxide on the individual particles that act as a barrier and that could not be broken during the melting trial. This barrier was not a problem for the particles in the lower part of crucible due to higher intensity of the electromagnetic forces in this area, as the copper in the upper part had also been melted and a portion had moved down to the lower part. Moreover, the load from the materials from the upper part of the crucible charged to those in the lower part and higher local temperature in the bottom of crucible than the upper part contributed to this.

The microstructural analysis of the agglomerated part showed the presence of three main phases (areas: 5, 6, 7 in Figure 10d). The dominant phase is a dark grey phase of a metallic matrix (Figure 10c), containing around 54.7 wt.% of Cu and 42.6 wt.% of Al as illustrated on area 5 in Figure 10d. Based on the EDS results (area 5 in Figure 10d) and considering the Al-Cu phase diagram [28], it is concluded that the chemical composition of this phase corresponds to the θ-CuAl_2_ phase. The results of microstructural analysis of the light grey phase (area 6 in Figure 10d) revealed that it was composed of an average of 72.5 wt.% Cu and 26 wt.% Al. According to the Al-Cu phase diagram [28], the chemical composition of the light grey phase corresponds to the ῃ′-CuAl phase. Area 7 in Figure 10c is a Si-rich phase containing around 98.4 wt.% of Si. The presence of the Si phase resulted from the chemical composition of the Al-Si-Mn alloy from the surface of which the Al dross sample used in experiment 1 was collected.

The overall chemical composition of the metallic portion in the agglomerated region shows that the Al content in this metal is much higher than that in the metal collected from the crucible’s bottom. This can be observed through the comparison of areas 5 and 6 (in the agglomerated part) with areas 1 to 3 in Figure 10. It may be concluded that the Al, Si, and Mn dissolution into the molten copper on the upper parts occurred due to the significant electromagnetic stirring of the copper and hence mass transport of these elements from the semi-solid dross into the liquid copper. However, the separation of the metallic particles and settling down to the crucible’s bottom did not occur. This is due to the contact of these metal droplets with the oxide part of the agglomerated material, which is a mixture of the metallic particles and nonmetallic material. The oxide network holds the metal droplets and prevents their separation due to the both gravity and the electromagnetic forces. In the lower part of the crucible, the same phenomena occurred; however, a rich copper metal was settled rapidly and its contact with the Al in the dross was prevented by the oxides present in the aluminum dross. This oxide layer over the metallic particles is seen in Figure 9. In short, a partial separation of Al through simple mixing of dross and copper followed by melting occurred. This process for better phase separation and Al recovery in the form of Cu-Al alloys can be improved through mechanical stirring or by applying alternative melting methods such as by using a rotary furnace for enhanced mixing.

#### 3.2.2. Flux-Aided Melting of Al-Rich Dross with Cu 

**Figure 11:**
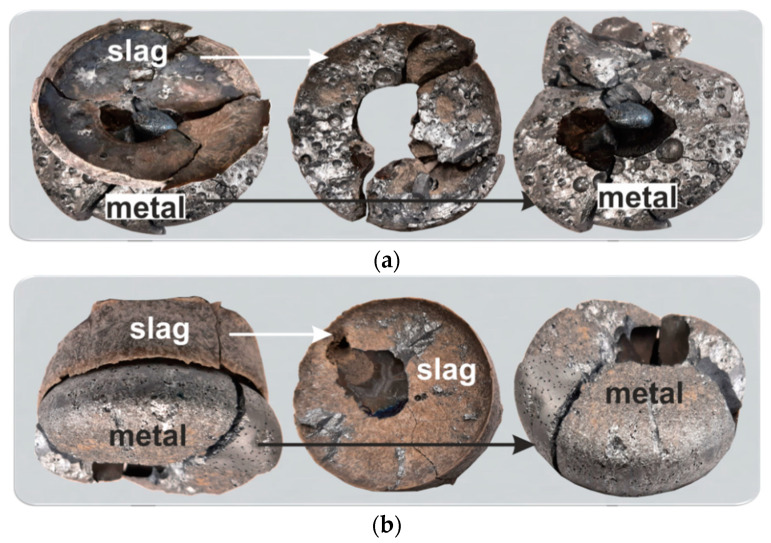
The samples after experiments: 2 (**a**) and 3 (**b**).

To overcome the challenges in metal separation through the simple induction melting described in Section 3.2.1, the slag-flux assisted melting experiments 2 and 3 were performed. The results for the melted Al dross with CaO-Al_2_O_3_ synthetic slags and Cu indicated that, in experiments 2 and 3, two complete separable metal and slag phases were obtained, as shown in Figure 11, and this approach in return was more successful than experiment 1. The metal phase was completely collected below the slag and the slag was easily separated from the metal due to no sticking between the two phases. 

**Figure 12:**
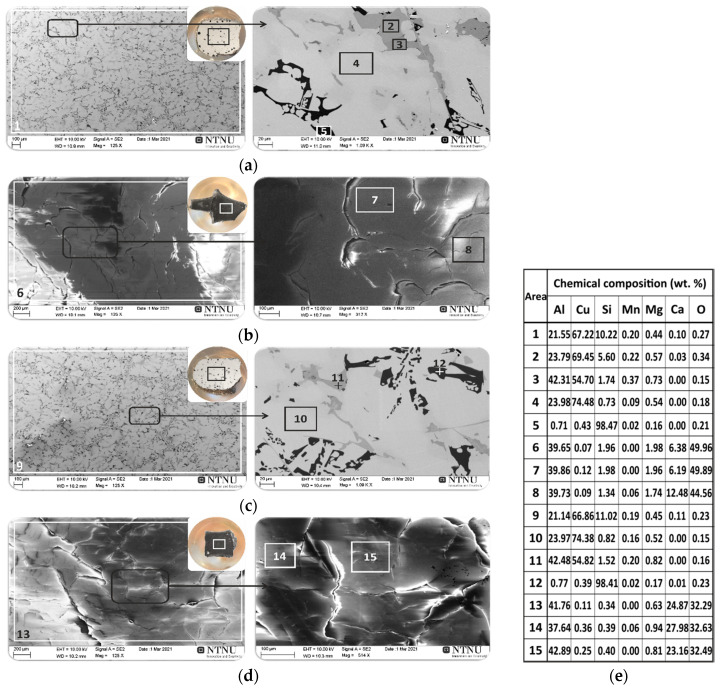
The SEM/EDS microstructural analysis of the cross-section of the metal produced in exp. 2 (**a**) and exp. 3 (**c**) and slag produced in exp. 2 (**b**) and exp. 3 (**d**); EDS (**e**).

The results of SEM/EDS microstructural analysis of the cross-section of the metals and slags produced in experiments 2 and 3 are shown in Figure 12. Both metals have very close chemical compositions and they are ternary Cu-Al-Si alloys with small amounts of Mg and Mn (areas: 1, 9 in Figure 12a,c respectively). The dominant phase is a light grey matrix (areas: 4, 10 in Figure 12a,c respectively), containing about 74 wt.% of Cu and 24 wt.% of Al. According to the Al-Cu phase diagram [28], the chemical composition of the dominant light grey phase corresponds to the Al_3_Cu_4_ phase. The microstructural analysis of the dark grey phase revealed that it was composed of an average of 54.7 wt.% Cu and 42.4 wt.% Al (areas: 3, 11 in Figure 12a,c respectively). Considering the Al-Cu phase diagram [28], it is concluded that the chemical composition of the dark grey phase corresponds to the θ-CuAl_2_ phase. In the microstructure Si-rich phase with about 98.4 wt.%, Si (areas: 5, 12 in Figure 12a,c respectively) was also found as a secondary precipitate, originated from the dross.

**Figure 13:**
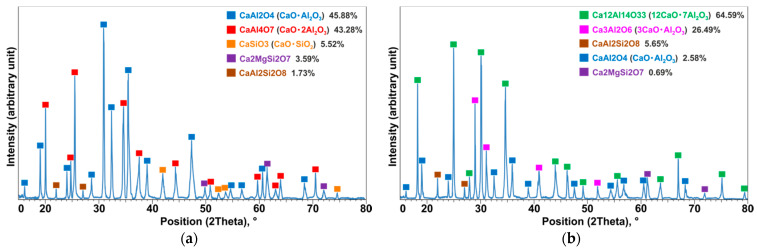
XRD patterns of the slags product in experiments: 2 (**a**) and 3 (**b**).

The results of elemental analysis of the produced slags indicated that in both cases the dominant phases were calcium aluminates (Figure 12b,d). In experiment 2, in which the proportion of oxides in the slag was CaO/Al_2_O_3_ = 2 (Table 1), the chemical composition of the produced slag contained an average of 39.8 wt.% Al, 9.3 wt.% Ca and 47.2 wt.% O (areas 7, 8 in Figure 12b), which suggests the presence of the CaAl_2_O_3_ phase. The XRD analysis of the slag samples confirmed these results according to the characterized phases in the XRD spectrums shown in Figure 13a. A semi quantitative XRD analysis indicated that about 46% of the material was CaO∙Al_2_O_3_ and 43% was CaO∙2Al_2_O_3_ phase as the dominant components in slag 2, which agrees with the CaO-Al_2_O_3_ binary phase diagram [48,49]. These findings confirmed the SEM/EDX results above and, in addition the XRD analysis, also showed some minor calcium silicate (CaSiO_3_), which is anticipated in the slag.

In the case of experiment 3, in which the CaO content was higher with CaO/Al_2_O_3_ = 4 (Table 1), the chemical composition of the produced slag contained on average 40.2 wt.% Al, 25.6 wt.% Ca and 32.5 wt.% O (areas 14, 15 in Figure 12d), suggesting the presence of the CaAl_2_O_3_ intermetallic phase. A semi quantitative XRD analysis (Figure 13b) indicated that about 64.6% of the material was 12CaO·7Al_2_O_3_ and 26.5% was 3CaO·Al_2_O_3_ phase as the dominant components, which agrees with the results of the EDS (areas 14,15 in Figure 12d) and the CaO-Al_2_O_3_ binary phase diagram [48,49]. The minor CaAl_2_Si_2_O_8_ phase was also identified in an amount of around 5.6%. Comparing the compositions of CaO and Al_2_O_3_ in the produced slags 2 and 3 indicates that the chemical and structural properties of the calcium-aluminate slag by-products can be controlled in this process and this can be beneficial for further use of the slags.

### 3.3. Metal Recovery and Dross Characterization

To evaluate the obtained results above, the metal recoveries for the experiments were calculated by mass balances. The known masses of the reactant and product materials and their known/measured concentrations were used. Obviously, the Cu recovery was 100% as it was in metallic form and it is noble compared with Al. Moreover, if Cu in the raw materials was in the form of oxide, its recovery was almost complete as the aluminothermic reduction of copper oxides occurred.

When CaO-Al_2_O_3_ slag was used as the flux, the Al_2_O_3_ of the dross ended up in the final slag. The amount of Al_2_O_3_ in the dross phase could be determined using the final slag composition and mass, and consequently the total metallic Al in the dross and Al recovery could be determined. In short, for the exp. 1 scenario, in which no synthetic slag was used, the Al recovery was only at the level of 39.1%, estimating that the Cu-Al alloy in the agglomerated part was about 40%.

Regarding the distribution of metallic Al of the dross into the produced alloy and separation of Al_2_O_3_ dross into the slag phase, the concentration of metallic Al in the dross can be determined for the slag-flux aided experiments by the following equation:

(2) $$\mathrm{metallic}\;\mathrm{Al}\;\mathrm{in}\;\mathrm{dross}\;\left(\%\right)=\left(\frac{\mathrm{mass}\;\mathrm{ofAl}\;\mathrm{in}\;\mathrm{produced}\;\mathrm{alloy}}{\mathrm{total}\;\mathrm{mass}\;\mathrm{of}\;\mathrm{Al}\;\mathrm{dross}\;}\right)\times 100$$

Moreover, Mn in dross will end up completely in the metal product as studied previously [7], while Si in dross, which is more metallic, will end up in both metal and slag phases, hence the amount of these elements in the dross can be determined as:

(3) $$\mathrm{metallic}\;\mathrm{Mn}\;\mathrm{in}\;\mathrm{dross}\;\left(\%\right)=\left(\frac{\mathrm{mass}\;\mathrm{of}\;\mathrm{Mn}\;\mathrm{in}\;\mathrm{produced}\;\mathrm{alloy}}{\mathrm{mass}\;\mathrm{of}\;\mathrm{Al}\;\mathrm{dross}\;}\right)\times 100$$

(4) $$\mathrm{metallic}\;\mathrm{Si}\;\mathrm{in}\;\mathrm{dross}\;\left(\%\right)=\left(\frac{\mathrm{mass}\;\mathrm{of}\;\mathrm{Si}\;\mathrm{in}\;\mathrm{produced}\;\mathrm{alloy}+\mathrm{slag}\;}{\mathrm{mass}\;\mathrm{of}\;\mathrm{Al}\;\mathrm{dross}\;}\right)\times 100$$

**Figure 14:**
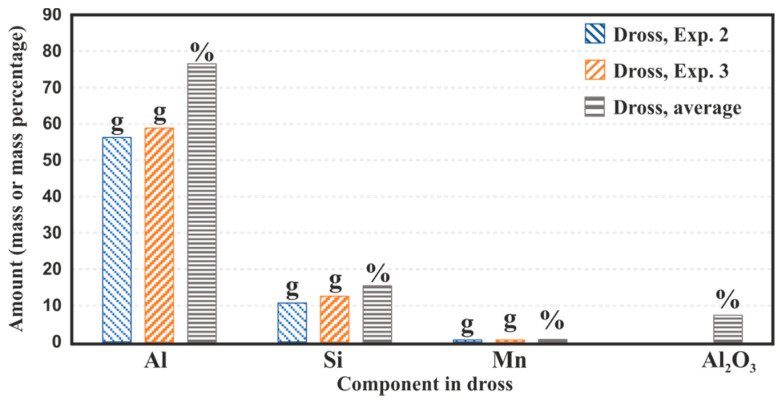
The determined amounts of dross components by mass balance by results of experiments 2 and 3.

The amounts of Al, Mn and Si in the utilized dross samples for the two experiments 2 and 3 were determined by mass balance, considering the masses of reactants and products, and also the chemical compositions of the products. It is worth noting that the amounts of the Si in the slags were determined based on the masses of the produced slags and the concentration of the Si-containing phases in Figure 13. The calculated masses of the elements in the dross in experiments 2 and 3 are presented in Figure 14. The data in this figure show that the utilized dross had about 76.5% Al, 15.4 % Si and 0.7% Mn, and the non-metallic components were mainly Al_2_O_3_, about 7.3%. The existence of carbide and nitrides in the dross was not detected by the XRD analysis and this is a fair approximation to consider Al_2_O_3_ as the main non-metallic component. The high concentration of Si in the dross was due to the existence of Si particles in the dross not being melted during aluminum alloying in the cast house.

### 3.4. Process Evaluation

**Figure 15:**
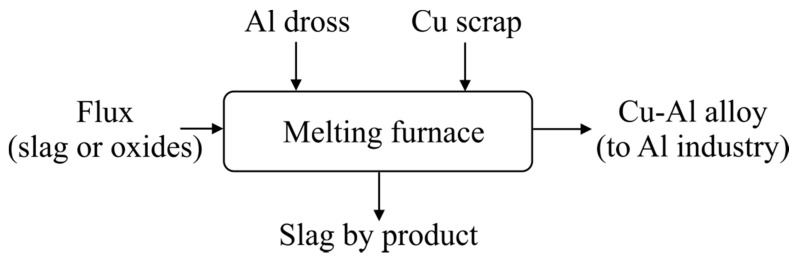
An illustration of the proposed process for Al dross valorization with Cu to recover metallic Al.

The recovery of metallic Al from the AWD was experimentally studied and it was shown that the addition of copper provided better conditions for the Al recovery. Moreover, the process was enhanced via the application of a synthetic slag flux and high-temperature treatment, providing better conditions for the separation of the molten metal and slag phases. The copper use and its mixing with metallic aluminum of the dross yielded an alloy with significantly higher density than Al, which provided better conditions for the physical separation of the produced metal from the slag in molten state. Therefore, we propose an integrated salt-free process for Al recovery from Al dross to produce Cu-Al alloys for further use or, in another word, Al recycling, where the Cu-Al alloys are used for making specific Al grades. The simplified process is illustrated in Figure 15.

The proposed process here has several advantages as compared with the existing treatment process for the AWD. Firstly, it is a simple single stage process in which the materials are melted. It is possible to use a small amount of commercial calcium aluminate slags and skip slag making, or instead of this type of slag utilization, only CaO (as the flux) is used. When CaO is used in the melting process, the Al_2_O_3_ of the dross reacts with the added CaO and yields the calcium aluminate slag. The volume of slag produced per unit mass of the metal product depends on the charged materials’ characteristics and quantities. This process is also sustainable compared with the salt-based processes, as there is no use of salt and the related precautionary steps that must be considered in materials handling and landfilling. Considering the fact that prevention of waste generation is always better than its valorization, the proposed dross treatment method in this research with no dangerous waste production is an important highlight. On the other hand, different compositions of the Cu-Al alloys can be produced, and they could be potentially utilized in the Al industry to produce the commercial grades of Al that contains Cu as an alloying element. In addition, Cu scrap or even copper oxide can be used in this process; however, the use of copper oxides may yield lower Al recovery. As we observed, if the utilized dross contained Si and Mn elements, the produced alloy contained these elements as well. Hence, the process can be integrated into Al production plant to recycle metallic components of a dross into the process for the alloy production. The produced slag byproduct is consumable and can be used in other industries such as steelmaking, the cement industry, and even the alumina industry as a feedstock for metallurgical alumina production [50,51].

## 4. Conclusions

The Al recovery from white dross by the remelting with the addition of Cu with and without oxide flux addition was studied. Moreover, the wettability of Al dross by Al-Cu-Si alloy was experimentally investigated. The main conclusions of the present work can be summarized as:

1. The temperature change in the range of 973–1373 K did not affect the wettability of the Al-Si-Cu alloy/Al dross system; the couple was a non-wettable system with a contact angle in the range of 125–130 degrees.
2. The melting of Al white dross and Cu particles yielded (I) an agglomerated fraction of metal and oxide in which the metallic droplets encapsulated in the oxide layer and (II) a completely melted/separated Cu-Al alloy settled under the agglomerated portion.
3. The addition of an oxide flux (melted calcium aluminate slags in this study) into the dross and copper mixture led to the complete separation of the metallic and non-metallic components of the dross.
4. In flux-assisted melting of dross and copper, the characteristics of the produced slag depend on the added flux properties and it was found that the phases in produced slag are dependent on the CaO/Al_2_O_3_ ratio and they can easily be engineered.
5. The applied experimental procedure in the present study can be applied to characterize Al dross through the separation of metallic components into a Cu-Al alloy and non-metallic components into a slag phase.
6. A process for Al dross and Cu slag valorization was introduced, which is sustainable and provides onsite recycling of Al from the dross.

## Figures and Tables

**Table 1:** The charge mixture details.

Exp. Number	Al Dross (g)	Cu Metal (99.99%) (g)	Synthetic Slag (g)	
			CaO	Al_2_O_3_
1	16.2	24.26	-	-
2	75.17	150.13	16.87	8.40
3	75.19	150.05	20.26	5.06

## Data Availability

Data available in a publicly accessible repository that does not issue DOIs. Publicly available datasets were analyzed in this study. This data can be found here: https://www.ntnu.edu/metpro (accessed on 23 July 2021).
